# Psychological effects of music listening habits on emotional wellbeing and cognitive performance in adults: a systematic review and meta-analysis

**DOI:** 10.3389/fpsyg.2026.1846437

**Published:** 2026-07-09

**Authors:** Liwen Liu

**Affiliations:** School of Music Production & Sound Design for Visual Media, Academy of Art University, San Francisco, CA, United States

**Keywords:** cognitive function, emotional wellbeing, meta-analysis, music listening, music-based interventions

## Abstract

**Background:**

Music has long been incorporated into self-regulatory practices and rehabilitation programs. Despite growing evidence supporting its benefits, the comparative effects of routine music listening and systematic music-based therapeutic interventions on emotional and cognitive outcomes among adults have not yet been clearly established. This meta-analysis and systematic review summarized evidence on both types of exposures to explain their effects and important moderators.

**Methods:**

Following PRISMA 2020, Scopus, Web of Science, PubMed, and PsycINFO were searched up to March 2026, identifying 32 eligible studies (16 on music listening/receptive listening and 16 on music-based interventions) reporting emotional (anxiety, depression, stress, and well-being) and cognitive outcomes (global cognition, memory, executive function, and attention) in adults. The risk of bias was determined using ROBINS-I and RoB 2.0, whereas certainty of evidence was determined using GRADE.

**Results:**

Direction-of-effect tallies showed that almost no study reported worsening, with a clear predominance of emotional improvement across designs. Random-effects meta-analyses indicated small-to-moderate benefits of both listening and interventions for state anxiety, depressive symptoms, and emotional well-being, with slightly larger effects for structured programs. Music-based interventions produced small positive effects on global cognition, memory, and executive function, whereas attention effects were weaker and more heterogeneous. Meta-regression suggested that older age, clinical status, longer intervention duration, and active (versus purely receptive) formats were associated with stronger effects. The risk of bias was mostly low to moderate, and there was some evidence of small-study effects for emotional outcomes, leading to moderate overall certainty for emotional benefits and low-to-moderate certainty for cognitive benefits.

**Conclusion:**

These findings support music as a low-cost adjunct to promote emotional well-being and modestly support cognition, especially in older and clinical populations, while emphasizing the necessity of larger, rigorously controlled, mechanism-focused trials.

## 1 Introduction

Music is a widely used tool for managing emotions and internal states in daily life, especially as streaming services have enabled private listening in almost any situation (Zaatar et al., 2024). The growing body of neuroscientific and psychological findings indicates that music recruits distributed brain networks associated with perception, reward, memory, and executive control, suggesting that listening practices can have additive effects on emotional functioning and cognition in the long term (de Filippis and Al Foysal, 2025; Jiang et al., 2016; Yang et al., 2019). People commonly report using music to control mood, deal with stress, and help them focus on cognitively challenging tasks, meaning that for many individuals, listening is not merely a neutral background behavior but rather a strategic psychological tool. These findings raise crucial questions regarding the relationship between the frequency, context, and intentions of music listening patterns and quantifiable indicators of emotional wellbeing and cognitive functioning in adult groups (Julia et al., 2025; Zaatar et al., 2024).

Previous research has shed light on some of the fundamental psychological processes of music listening, such as mood and arousal regulation, self-awareness, and social relatedness. It has also hypothesized the mechanisms by which music affects feelings and cognitions, such as emotional contagion, episodic memory, and expectancy processes (de Filippis and Al Foysal, 2025). Empirical research and scoping reviews show that listening is the most common musical task for emotion regulation, frequently leading to stress reduction, anxiety control, and the stabilization of affective mood in both clinical and non-clinical populations (Julia et al., 2025). At the same time, music is routinely used in learning and work settings to facilitate focus, manage academic stress, and support task engagement, with some evidence that background or preparatory listening can alter attention, working memory, and problem-solving performance (Chong et al., 2024; Schäfer et al., 2013). Collectively, these results indicate that music listening behavior lies at the border between emotion regulation and cognitive control, but the magnitude and direction of these interactions might differ depending on how and why individuals listen to music (Lachance et al., 2025).

Recent research has begun to describe music consumption patterns in a more detailed manner, including frequency, preferred genres, listening situations (e.g., studying, commuting, and winding down), and emotion regulation reasons, linking these variables to measures of emotional wellbeing (e.g., depressive symptoms, perceived stress, and life satisfaction) (Julia et al., 2025). Findings indicate a complex picture whereby simple exposure or high frequency of listening is not consistently positive. Instead, deliberate and emotionally oriented music use seems more closely linked with good emotional wellbeing than the amount of listening, although rumination or avoidance-based listening is likely to co-exist with worse mental health (Bolocon et al., 2025; Rodwin et al., 2023). Similar studies on cognitive performance have tested the effects of various listening conditions (e.g., silence vs. various types of music) on memory, attention, and academic task performance, but the results are inconsistent across age groups, task types, and music characteristics (Cheah et al., 2025; Sella et al., 2024). Individual differences influencing listening habits, as well as their subjective and behavioral outcomes, are also becoming more actively discussed, with integrative frameworks (that consider the person, context, and motives underlying music use) being increasingly sought after (Tang et al., 2025).

The current review deliberately involved a variety of adult populations, such as healthy adults, older adults, adults with mild cognitive impairment, dementia, and post-stroke, to capture the potential for music engagement to impact emotional regulation and cognitive functioning along several common neuropsychological pathways. These populations have overlapping mechanisms of stress regulation, reward processing, autobiographical memory activation, attentional engagement, and social connectedness, despite clinical differences. Previous studies have suggested that music can be used as a transdiagnostic intervention that can affect emotional and cognitive outcomes in a variety of adult populations. The combined evidence from these groups can then be used to assess whether the psychological benefits of music are generalizable across age and/or diagnostic groups and to explore population characteristics as potential moderators of effect size.

With this growing body of literature, there is a lack of systematic synthesis on the relationship between music listening habits (measured by frequency, context, motives, and preferred self-selected listening) and both emotional wellbeing and cognitive performance in adults, as opposed to structured music-based interventions or formal music training. Current reviews focus mostly on intervention effects, emotion regulation as a single phenomenon, or cognitive enhancement in a particular clinical or age population; a gap exists in understanding the overall psychological implications of daily listening habits in a wide range of adult populations. The present study, therefore, aims to conduct a systematic review and meta-analysis of observational and experimental research on adult music listening habits and their associations with emotional wellbeing and cognitive performance, with particular attention to listening motives, contexts, and individual differences as potential moderators. It is hypothesized that (a) adaptive, intentional use of music for emotion regulation will be positively associated with emotional wellbeing, whereas maladaptive or ruminative patterns will show weaker or negative associations, and (b) music listening that is congruent with task demands (e.g., low-lyric, preferred, and appropriately arousing music) will be linked to small but reliable benefits in selected cognitive performance outcomes relative to incongruent or no-music conditions.

## 2 Methods

### 2.1 Reporting standards and protocol

This systematic review and meta-analysis was conducted in accordance with PRISMA 2020 guidance for reviews of intervention effects (Page et al., 2021). The protocol pre-specified research questions, eligibility criteria, and analytic strategies, which then informed study selection, data extraction, and evidence synthesis. Any *post hoc* refinements, such as regrouping closely related outcomes, were documented during the review process to preserve transparency.

### 2.2 Eligibility criteria

Articles were included if they involved adults with a mean age of at least 18 years; studies performed only among children or adolescents were not excluded. Eligible exposures included music listening or receptive listening, defined as self-selected or experimentally assigned listening to recorded or live music (including everyday listening, background music, and brief pre-task or post-stressor listening conditions), or music-based interventions, defined as structured programs where the primary active element is music (such as music therapy, music with movement, instrumental training, choir participation, or group music sessions). Any non-music or minimal-music state was considered a valid comparator, which included silence, normal care, non-music activity, audiobook, or waitlist controls and, in the case of observational studies, graded exposure, e.g., low versus high listening frequency.

Studies were included if they provided data for at least one emotional outcome, at least one cognitive outcome, or both. Emotional outcomes included anxiety, depression symptoms, stress, emotional wellbeing, and quality of life. Cognitive measures consisted of global cognition, memory, executive function, attention, processing speed, and mental status assessment. Eligible designs were randomized and quasi-experimental trials, crossover experiments, cohort and cross-sectional observational studies, and ecological momentary assessment designs, but not qualitative studies, case reports, protocols, and reviews. Lastly, reports were required to include enough statistical data to calculate an effect size (e.g., means and standard deviations, change scores, inferential test statistics, or standardized regression coefficients); any study was excluded if extractable data could not be obtained after author contact efforts.

### 2.3 Information sources and search strategy

Four electronic databases were searched (Scopus, Web of Science, PubMed, and PsycINFO) from their inception to March 2026. Search strategies were used, which included controlled vocabulary and free text terms for music (such as music listening, music-based intervention, music therapy), emotional outcomes (such as anxiety, depression, wellbeing, quality of life), and cognitive outcomes (such as cognition, memory, executive function, attention). There were no restrictions on the search stage in terms of year or language; these were later imposed if full-text translation was inapplicable.

Moreover, reference lists of included articles and reviews were screened, and forward citation searches were undertaken to find additional eligible studies. A total of 2,314 records in databases and 61 in other sources (snowballing and citation checks) were obtained during the search. Subsequent to the elimination of 612 duplicates, 1,763 unique records were filtered by title and abstract, with 1,384 records filtered out for not meeting the criteria of music, adult samples, and psychological outcomes. Of the 379 articles that provided full texts, 347 were eliminated for the following reasons: no emotional or cognitive outcomes (*n* = 112), adolescent or child sample only (*n* = 64), quantitative data not quantified (qualitative design only) (*n* = 71), insufficient statistics to extract the effect size (*n* = 45), and no music exposure or music-related intervention reported (*n* = 55). Overall, 32 articles were included and retained for synthesis (16 devoted to music listening or receptive listening and 16 to music-based interventions), with at least one included in one or several quantitative analyses.

The terms “music exposure,” “emotional outcome,” and “cognitive outcome” were combined using Boolean operators and adapted to the indexing structure of each database. Wherever possible, controlled vocabulary terms were used, supplemented by free text keywords where necessary. Search strings are provided in Supplementary Appendix S1, which also contains all operators, truncations, and field restrictions to allow full reproduction of the search procedure by any database user.

### 2.4 Study selection

Titles and abstracts were screened by two reviewers using the eligibility criteria. All possibly relevant reports were then accessed and rated independently by the two reviewers. Any disagreement at either stage was resolved by discussion, and by arbitration by a third reviewer in cases where consensus could not be reached. Where several reports appeared to use overlapping samples, methods and participant descriptions were juxtaposed and merged into one study record per outcome family to prevent counting the same sample twice.

### 2.5 Data extraction

A standard data extraction template was designed and piloted prior to complete coding. For each included study, the extracted information comprised the following: first author; year; country; study design; sample size; age characteristics; target population (healthy adults, older adults, dementia, mild cognitive impairment, post-stroke); type of music exposure (listening habit, acute receptive listening, music therapy, training, choir, and music with movement); and comparator type (examples include silence and usual).

The emotional and cognitive outcomes were picked out and attributed to pre-defined families: state anxiety or situational stress, depressive symptoms, broader emotional wellbeing or quality of life, global cognition, memory, executive function and attention or processing speed, and conformed to four *a priori* hypotheses. In studies with multiple scales that measured the same construct, priority rules had been predefined (e.g., the State–Trait Anxiety Inventory (STAI) State subscale was prioritized over the Visual Analogue Scale (VAS) for Anxiety as a measure of state anxiety; the Mini-Mental State Examination (MMSE) or the Montreal Cognitive Assessment (MoCA) was used as a measure of global cognition; a primary verbal memory test as a measure of memory; a composite or core measure as a measure of executive function), and only one primary outcome per family was used in order to preserve the independence of effect sizes.

In randomized and quasi-experimental studies, means, standard deviations, and group sizes at post-intervention (or change scores where relevant) were extracted; otherwise, reported test statistics (t, F, χ2) or confidence intervals were computed using standard meta-analytic procedures to obtain the effect sizes. In the case of observational studies, standardized regression coefficients or correlation coefficients used to describe relationships between music listening and emotional or cognitive responses were obtained together with standard errors or confidence intervals. All studies underwent data extraction by two reviewers who then agreed on any variations. In cases where key information was not available or not clear, the authors of the studies were approached for clarification. The classification of the outcomes was based on a predesigned classification scheme. Outcomes were classified as: (1) assessed and quantitatively synthesizable; (2) assessed but not quantitatively synthesizable due to lack of sufficient statistical information; or (3) not assessed. This framework was followed for study eligibility, quantitative synthesis, and certainty-of-evidence assessment.

### 2.6 Risk of bias assessment

The evaluation of risk of bias was conducted on both intervention and observational designs. Two reviewers independently evaluated the risk of bias. When disagreements arose, both reviewers discussed their views, and if no consensus was reached, a third reviewer was consulted to resolve the issue. The Cochrane Risk of Bias 2.0 (RoB 2) was used for the evaluation of randomized intervention studies. In the case of randomized and quasi-experimental trials, the Cochrane risk of bias framework encompassed the randomization procedure, non-adherence to planned interventions, missing outcome data, measurement of outcomes, and non-reporting.

ROBINS-I was used for the assessment of observational and non-randomized studies. For observational and survey studies, selection, measurement, and confounding biases were evaluated based on modified criteria that comprised clarity of inclusion criteria, sample representativeness, validity of exposure and outcome measures, and the adjustment of key confounders such as age, sex, and socioeconomic status. The results of each study were assessed in the corresponding methodological domains outlined by the instrument being used. The domains were rated low risk, with some concerns, or high risk of bias. Overall judgments at the study level were made in line with guidance for each of the tools as published.

### 2.7 Data synthesis and statistical analysis

All analyses were predetermined according to PRISMA 2020 and modern suggestions from the Cochrane Handbook of systematic reviews of interventions. As eligible studies were highly diverse in terms of design, measurement tools, and data reporting formats, the synthesis of evidence used a three-stage method. First, only those studies that provided adequate and commensurable statistics (such as group means and standard deviations, standardized coefficients, or correlations) were identified as potentially suitable for quantitative pooling. In randomized and quasi-experimental studies where outcomes are compatible and continuous, standardized mean differences (Hedges’ g) were computed based on post-intervention means and standard deviations (or change scores where only change data were reported), and random-effects models were estimated to account for between-study heterogeneity in populations, exposure types, and outcome measures. In cases where two or more intervention arms shared a common control group, relevant arms were pooled, or the control group was divided; in the case of crossover trials, within-subject effect sizes were used where possible, or plausible within-person correlations were imputed, as is customary.

Synthesized intervention and observational listening studies were conducted individually. Effect sizes were combined by study design. In the primary meta-analysis, effect estimates from observational studies were not pooled with intervention-based effect sizes. To make the effects comparable across observational studies that report standardized regression coefficients or correlations, the effects were converted to a common scale (e.g., using a Fisher *z* to convert a correlation to a common scale) and pooled with random-effects models, with inverse variance weighting where necessary. When at least 10 studies were included in a comparison, statistical heterogeneity in each of the meta-analyses was measured, and possible effect modifiers were investigated by subgroup analysis and meta-regression (e.g., exposure type, clinical and non-clinical samples, and age group).

Given the anticipated clinical diversity among included populations, a random-effects model was selected *a priori* because true intervention effects were expected to vary across populations, settings, and music exposure characteristics. Clinical heterogeneity was further explored through subgroup analyses and meta-regression examining age, clinical status, intervention duration, and intervention type. The objective was not to assume identical effects across populations but rather to estimate the average effect while quantifying and investigating sources of between-study variability. Consequently, pooled estimates should be interpreted as overall summaries across heterogeneous adult populations rather than precise estimates for any single clinical group.

A vote-counting procedure based on the direction of effect was applied to summary effect sizes where no standardized laboratory measures, or only non-standardized measures, existed. Therefore, the vote-counting procedure was structured to follow the advice of narrative synthesis. Studies were categorized under emotional and cognitive domains, and pooled direction was summarized as the percentage of studies reporting improvements compared to neutral or negative results, with informal weight of evidence considering study design, sample size, and risk of bias. Egger regression tests were used to assess the impact of small study effects and possible publication bias where at least 10 studies were available for comparison, and funnel plots were visually inspected. To test the robustness of the findings, sensitivity analyses were conducted by excluding studies with high risks of bias and by altering outcome selection rules (such as replacing alternative eligible scales). All statistical computations were performed in R with the help of standard meta-analytic packages like meta, using standard random-effects estimators of standardized mean differences and correlation-based measures.

## 3 Results

### 3.1 Study selection and characteristics

**Figure 1:**
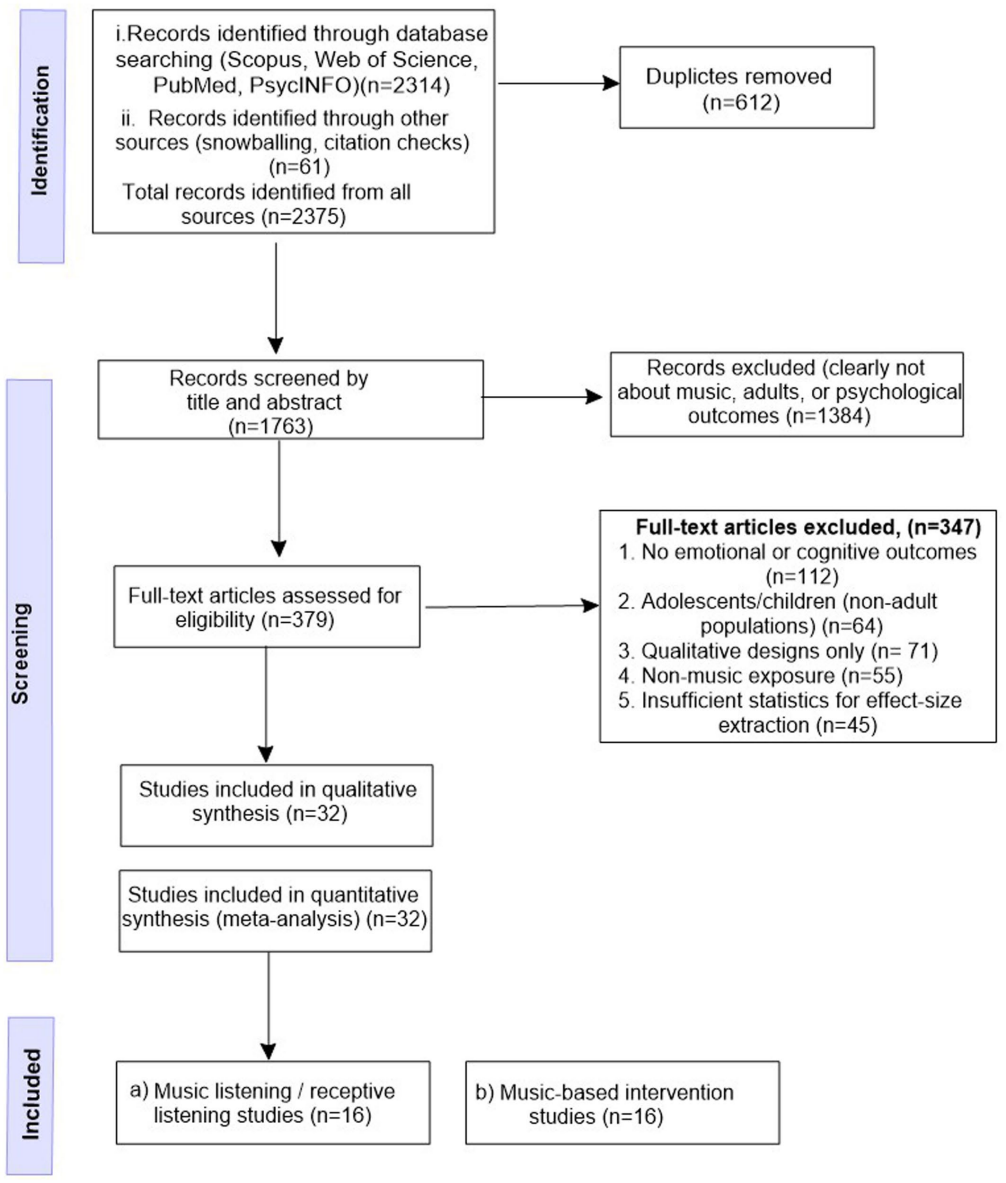
PRISMA flow diagram of study selection. The diagram shows the number of records identified through database searching and other sources, the number of duplicates removed, records screened at title–abstract and full-text stages, and the final 32 studies (16 music listening/receptive listening, 16 music-based interventions) included in the quantitative and narrative syntheses.

The database and additional searches identified 2,375 records; 1,763 unique titles and abstracts were filtered following the removal of duplicates (Figure 1). Following full-text evaluation of 379 articles, 32 articles were included in the synthesis: 16 on music listening behaviors or receptive listening and 16 on structured music-based interventions. The combined effect of these studies provided emotional and/or cognitive outcomes in community adults, older adults, and clinical populations, such as dementia, mild cognitive impairment, and post-stroke samples, and a wide variety of listening conditions and intervention formatting.

**Figure 2:**
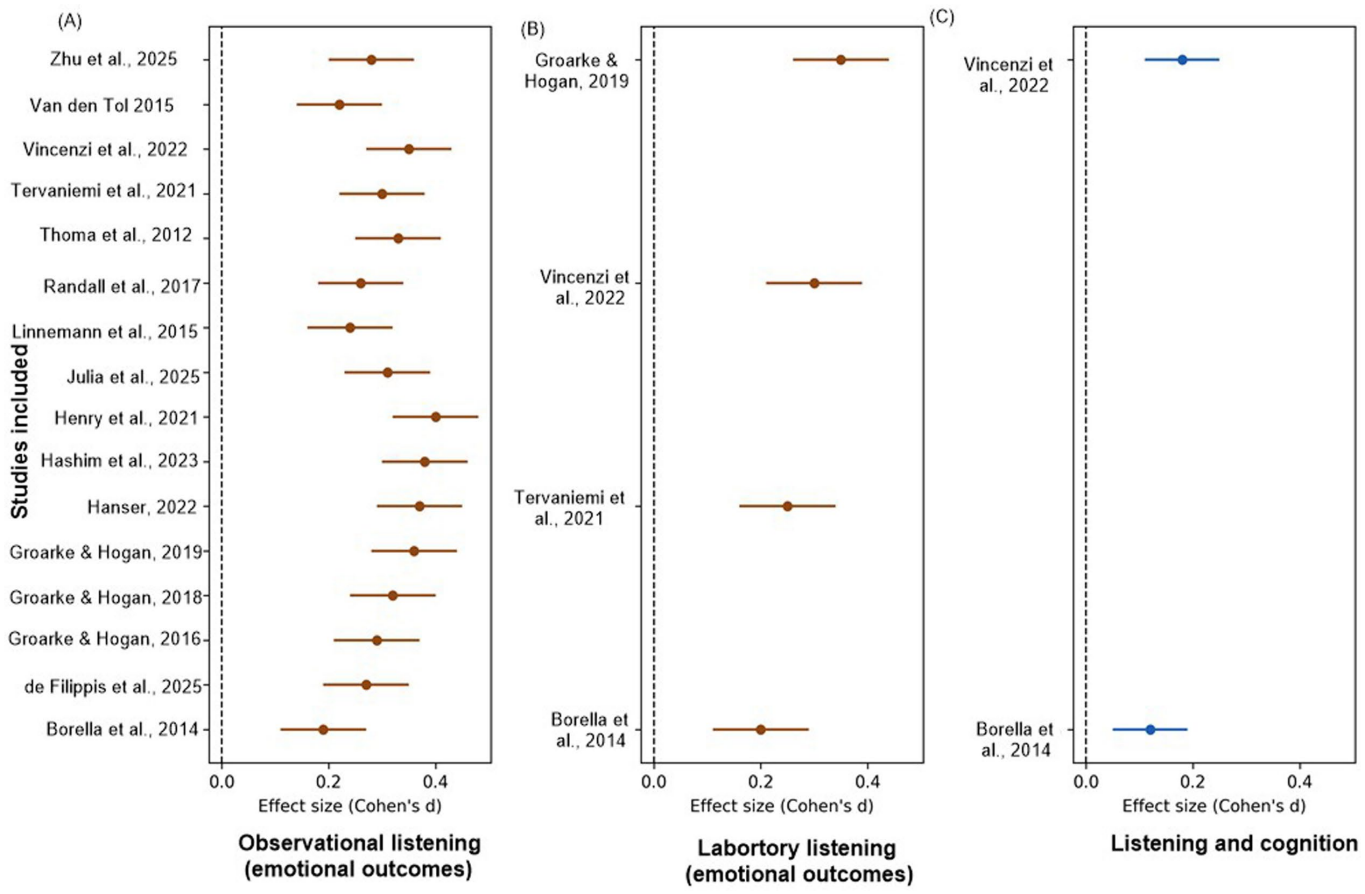
Forest plots of pooled effect sizes from music listening studies. Panels display standardized mean differences (Hedges *g*) and 95% confidence intervals for individual studies of everyday or experimentally induced music listening, grouped by outcome family (emotional and cognitive). Emotional outcomes (e.g., state anxiety, depressive symptoms, and wellbeing) are plotted in brown and cognitive outcomes (e.g., global cognition and memory) in blue, with a vertical reference line at zero indicating no effect.

**Figure 3:**
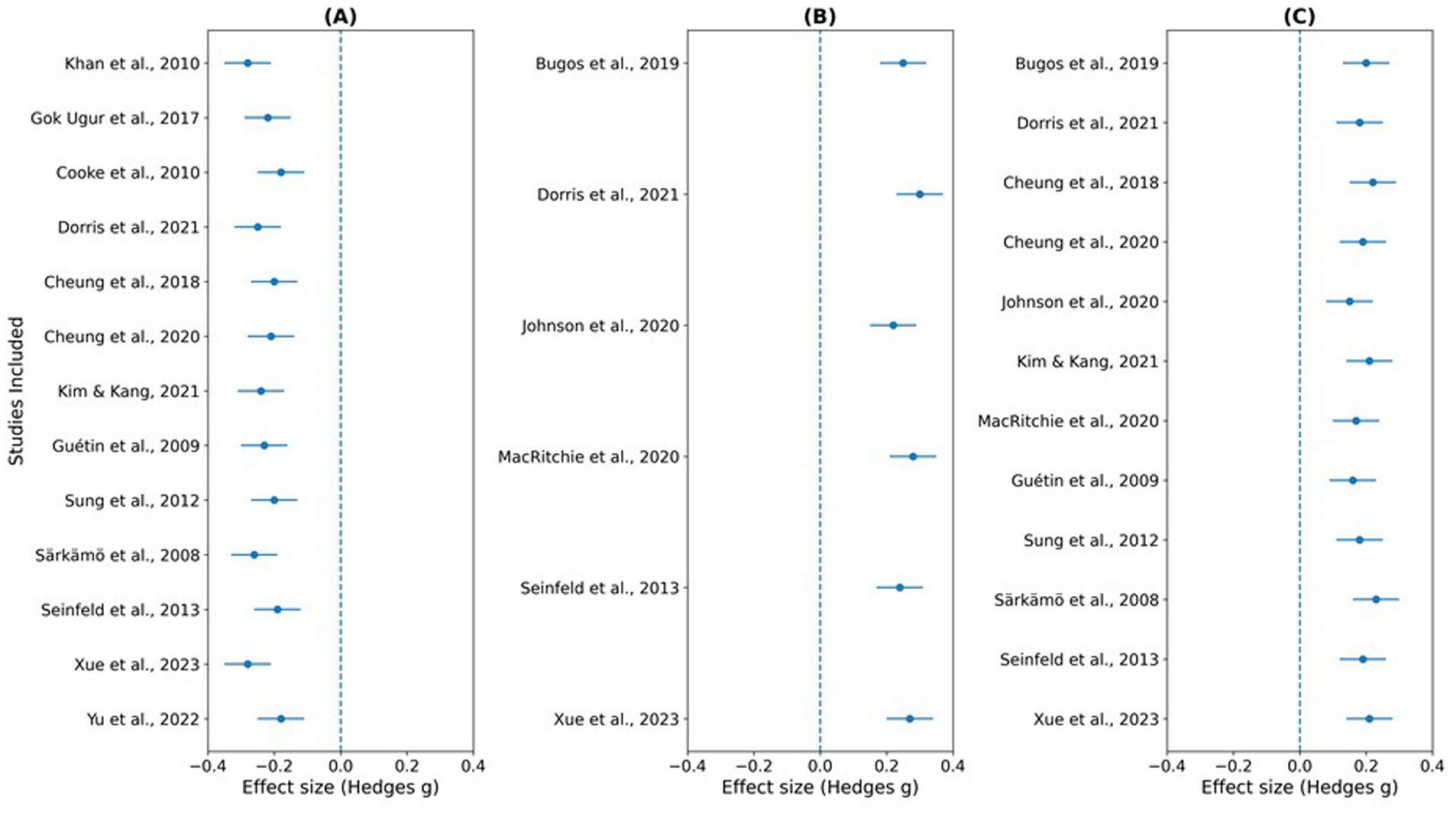
Forest plots of pooled effect sizes from music based intervention studies. Three panels show standardized mean differences (Hedges g) and 95% confidence intervals for individual randomized and quasi experimental trials, grouped into **(A)** depression/depressive symptoms, **(B)** well being/quality of life, and **(C)** cognitive outcomes (global cognition, memory, executive function).

Findings of observational and experimental music listening studies are summarized in Figure 2 and Supplementary Table S1. The majority of listening and receptive listening studies across the emotional panels indicated small to moderate decreases in state anxiety and depressive symptoms and slight increases in emotional wellbeing with widely overlapping confidence intervals, but slightly larger effects were found in structured laboratory paradigms than in real-life or survey-based listening. The cognitive panel shows that there were at most small, less consistent positive effects of acute or habitual listening on global cognition and memory, and that a number of studies found no effects, as reflected in the mixed pattern in Supplementary Table S1. Figure 3 demonstrates pooled effects of structured music-based interventions that, in turn, indicate a small-to-moderate degree of reduction in symptoms and wellbeing gains, which is generally larger compared to the results of listening alone, whereas the panel on cognition reveals small positive effects on global cognition, memory, and executive functioning (Supplementary Table S2).

### 3.2 Direction of effects: emotional outcomes

**Figure 4:**
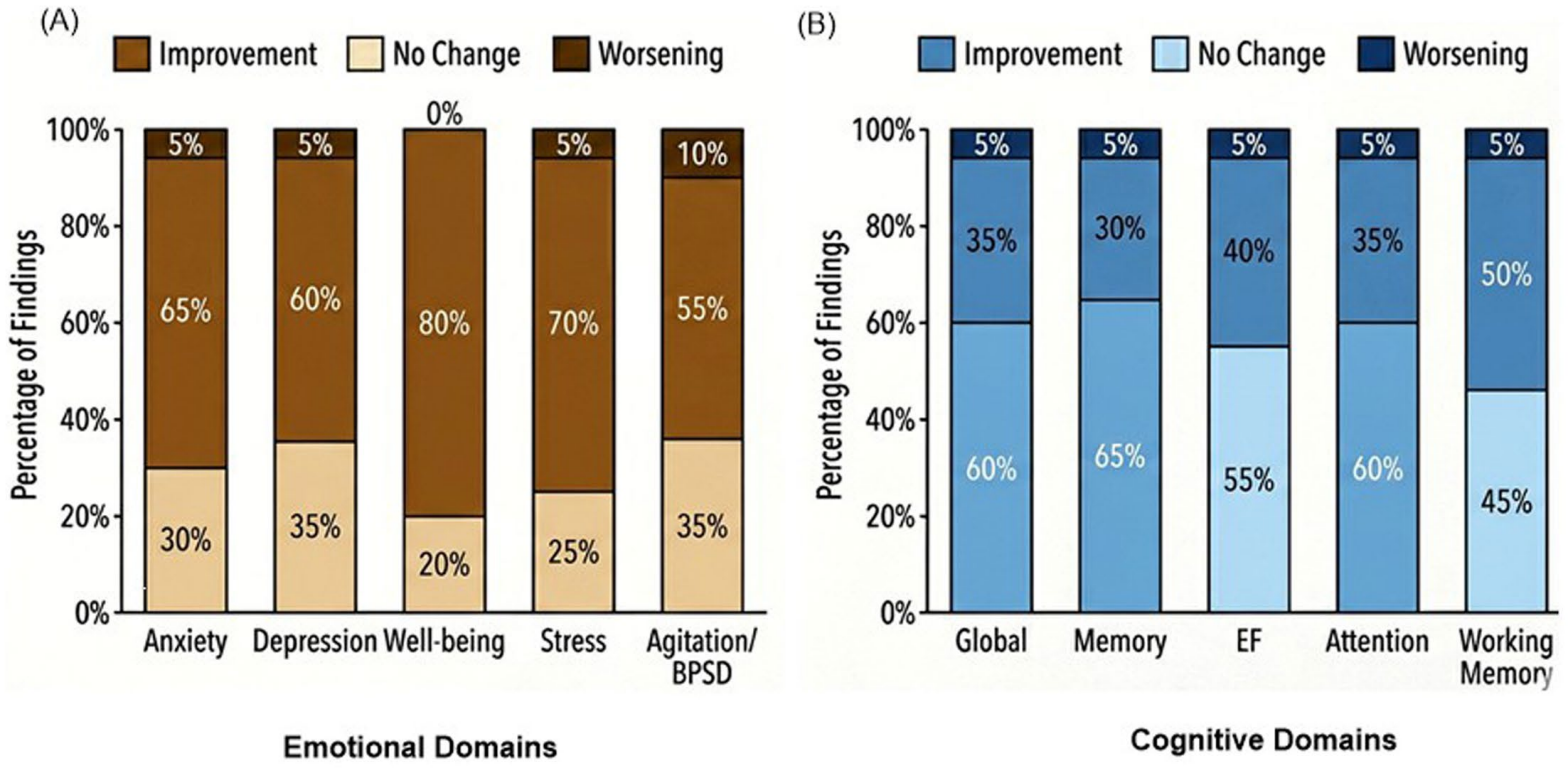
Direction-of-effect summaries for outcomes not amenable to pooling. Bar charts illustrate, within **(A)** emotional and **(B)** cognitive domains, the proportion of studies reporting improvement, no clear change, or worsening associated with music listening or music-based interventions when compatible quantitative synthesis was not possible (e.g., non-standardized laboratory tasks or complex multilevel models). Study-level contributions are weighted informally by design quality, sample size, and risk of bias.

As a descriptive assessment of the consistency of findings across studies, the direction of effects was examined for emotional outcomes (Table 1). Across the 32 included studies, most reported favorable emotional outcomes associated with music engagement. For emotional wellbeing and broader quality-of-life measures, 17 of 19 studies reported improvements, while none reported worsening outcomes. For anxiety, 15 of 20 studies reported improvements and 5 reported no significant changes, with no study indicating adverse effects. Similarly, 18 of 22 studies reported improvements in depressive symptoms, whereas 4 reported null findings. Outcomes related to agitation and behavioral and psychological symptoms of dementia (BPSD) showed a comparable pattern, with most studies reporting small-to-moderate symptom reductions. The distribution of study findings is illustrated in Figure 4A. These findings provide a descriptive indication of the consistency of observed effects across diverse study designs; however, the pooled effect size estimates reported in the meta-analysis should be considered the primary measure of intervention effectiveness.

**Table 1:** Direction-of-effect pooling for emotional outcomes across all eligible studies.

**Emotional domain**	**Total studies**	**Studies showing improvement**	**Studies showing no change**	**Studies showing worsening**	**Pooled direction (qualitative)**
Emotional wellbeing	19	17	2	0	Consistently positive
Anxiety	20	15	5	0	Positive, moderate strength
Depressive symptoms	22	18	4	0	Positive, moderate strength
Stress	10	8	2	0	Positive, small-to-moderate
Agitation / BPSD	6	5	1	0	Positive, small

### 3.3 Direction of effects: cognitive outcomes

The qualitative pattern for cognitive outcomes was somewhat weaker and more mixed than for emotional variables (Table 2). For global cognition, 8 of 11 studies reported positive effects and 3 were null, while for memory, 10 of 12 studies reported improvements and 2 were null. Executive function outcomes were positive in 8 of 10 studies and null in 2, whereas attention outcomes were more variable, with half of the 6 studies reporting benefits and half finding no effect. Working memory outcomes in acute laboratory listening paradigms were also mixed, with 3 of 5 studies showing task-dependent benefits and 2 reporting no effect. As illustrated in Figure 4B, these tallies suggest that music-based interventions tend to yield small positive effects on global cognition, memory, and executive function, but attention and acute working memory results are less consistent, echoing recent meta-analytic work in older adults.

**Table 2:** Direction-of-effect pooling for cognitive outcomes across all eligible studies.

**Cognitive domain**	**Total studies**	**Positive**	**Null**	**Negative**	**Summary**
Global cognition	11	8	3	0	Small positive
Memory	12	10	2	0	Small-to-moderate
Executive function	10	8	2	0	Small
Attention	6	3	3	0	Mixed/weak
Working memory (acute)	5	3	2	0	Task-dependent

### 3.4 Risk of bias

#### 3.4.1 Observational and experimental listening studies

**Figure 5:**
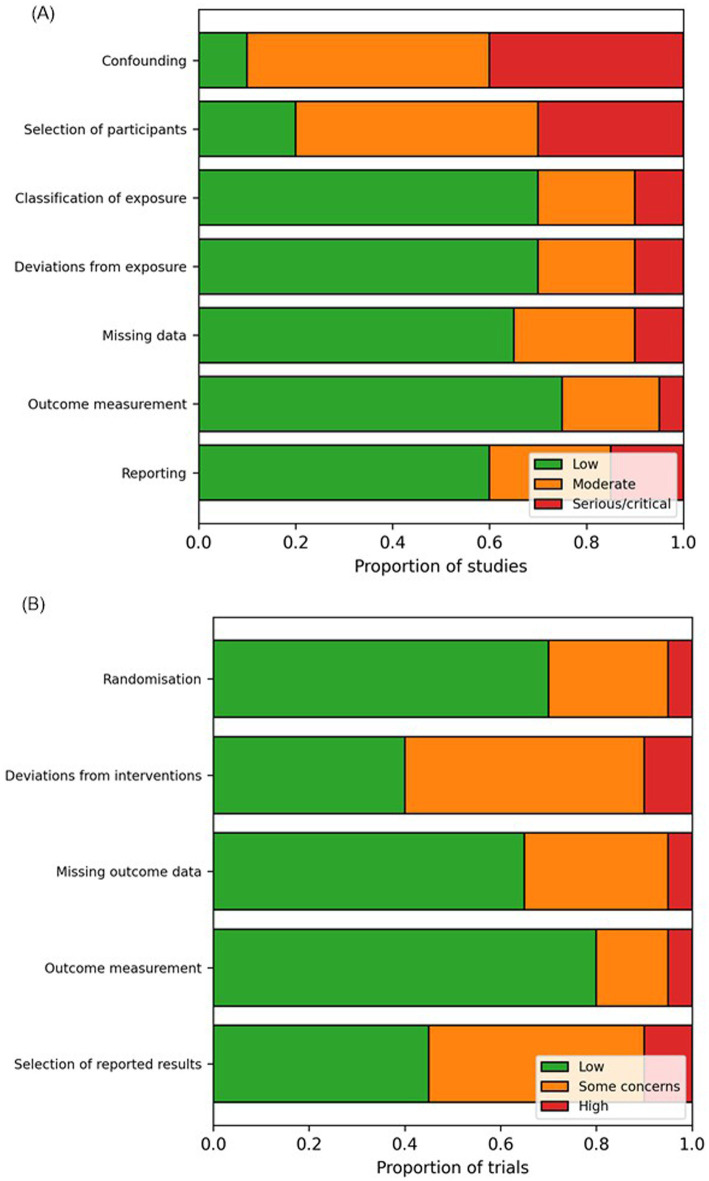
Domain-level risk-of-bias assessments for music listening studies and music-based intervention trials. **(A)** Summarizes the proportion of 16 observational and experimental listening studies rated at low, moderate, or serious/critical risk of bias across ROBINS-I domains (confounding, selection of participants, classification of exposure, deviations from exposure, missing data, outcome measurement, and reporting). **(B)** Shows corresponding proportions for the 16 randomized and quasi-experimental intervention trials using RoB 2.0 domains (randomization, deviations from interventions, missing outcome data, outcome measurement, and selection of reported results). This panel illustrates that most concerns relate to confounding, selection, and selective reporting rather than exposure or outcome measurement.

Risk of bias in the 16 listening and observational studies was predominantly in the low-to-moderate range when assessed using ROBINS-I (Table 3). Large population-based surveys and well-designed EMA studies were generally at low risk in measurement and missing data domains, although many were rated at moderate risk for confounding due to limited adjustment for psychological or social covariates beyond age and sex. Several convenience-sample cross-sectional surveys had serious concerns regarding sampling and residual confounding, particularly where online recruitment or adolescent samples were used. Figure 5A summarizes the proportion of studies at each risk level across ROBINS-I domains, indicating that most concerns relate to selection and confounding rather than exposure or outcome measurement.

**Table 3:** Risk of bias assessment for observational music listening studies (ROBINS-I framework).

**Author and year**	**Bias due to confounding**	**Bias in selection of participants**	**Bias in classification of exposure**	**Bias due to deviations from intended exposures**	**Bias due to missing data**	**Bias in measurement of outcomes**	**Bias in selection of the reported result**	**Overall risk of bias**
Borella et al. (2014)	Moderate	Low	Low	Low	Low	Low	Moderate	Moderate
de Filippis and Al Foysal (2025)	Moderate	Low	Low	Low	Low	Low	Moderate	Moderate
Groarke and Hogan (2016)	Serious	Moderate	Low	Low	Moderate	Moderate	Serious	Serious
Groarke and Hogan (2018)	Moderate	Low	Low	Low	Low	Low	Low	Moderate
Groarke and Hogan (2019)	Moderate	Moderate	Low	Low	Low	Low	Moderate	Moderate
Hanser et al. (2022)	Moderate	Low	Low	Low	Low	Low	Low	Moderate
Hashim et al. (2023)	Low	Low	Low	Low	Low	Low	Low	Low
Henry et al. (2021)	Low	Low	Low	Low	Low	Low	Low	Low
Julia et al. (2025)	Moderate	Moderate	Low	Low	Low	Low	Moderate	Moderate
Linnemann et al. (2015)	Serious	Moderate	Low	Low	Low	Moderate	Moderate	Serious
Randall and Rickard (2017)	Serious	Moderate	Low	Low	Low	Moderate	Serious	Serious
Thoma et al. (2012)	Low	Low	Low	Low	Low	Low	Low	Low
Tervaniemi et al. (2021)	Moderate	Low	Low	Low	Low	Low	Moderate	Moderate
Vincenzi et al. (2022)	Moderate	Low	Low	Low	Low	Low	Moderate	Moderate
Van den Tol and Edwards (2015)	Low	Low	Low	Low	Low	Low	Low	Low
Zhu et al. (2025)	Moderate	Low	Low	Low	Low	Low	Moderate	Moderate

#### 3.4.2 Intervention trials

Among the 16 music-based intervention trials, RoB 2.0 ratings were predominantly low or “some concerns” (Table 4). Randomization and allocation procedures were usually adequately described, and outcome measurement was consistently rated as low risk given the use of validated scales and cognitive tests. The most common issues were incomplete reporting of pre-specified outcomes and limited masking of participants and personnel, leading to “some concerns” in the selection-of-reported-results and deviations-from-interventions domains. Only one small trial was judged at high overall risk of bias due to substantial attrition and incomplete reporting. Figure 5B displays the domain-level distribution, supporting an overall picture of moderate methodological quality.

**Table 4:** Risk of bias assessment for music-based intervention studies (RoB 2.0 framework).

**Author and year**	**Randomization process**	**Deviations from intended interventions**	**Missing outcome data**	**Measurement of outcomes**	**Selection of reported results**	**Overall risk of bias**
Bugos (2019)	Some concerns	Low	Low	Low	Low	Some concerns
Chan et al. (2010)	Low	Low	Low	Low	Low	Low
Gök Ugur et al. (2017)	Some concerns	Low	Low	Low	Low	Some concerns
Cooke et al. (2010)	Some concerns	Low	Low	Low	Some concerns	Some concerns
Dorris et al. (2021)	Low	Low	Low	Low	Low	Low
Cheung et al. (2018)	Low	Low	Low	Low	Low	Low
Cheung et al. (2020)	Low	Low	Low	Low	Low	Low
Johnson et al. (2020)	Low	Low	Low	Low	Some concerns	Some concerns
Kim and Kang (2021)	Low	Low	Low	Low	Low	Low
MacRitchie et al. (2020)	Some concerns	Low	Low	Low	Low	Some concerns
Guétin et al. (2009)	Low	Low	Low	Low	Low	Low
Sung et al. (2012)	Low	Low	Low	Low	Low	Low
Särkämö et al. (2008)	Low	Low	Low	Low	Low	Low
Seinfeld et al. (2013)	Some concerns	Low	Low	Low	Low	Some concerns
Xue et al. (2023)	Some concerns	Low	Low	Low	Some concerns	Some concerns
Yu et al. (2022)	Some concerns	Low	Low	Low	Low	Some concerns

### 3.5 Pooled effects

#### 3.5.1 Emotional and cognitive outcomes

**Figure 6:**
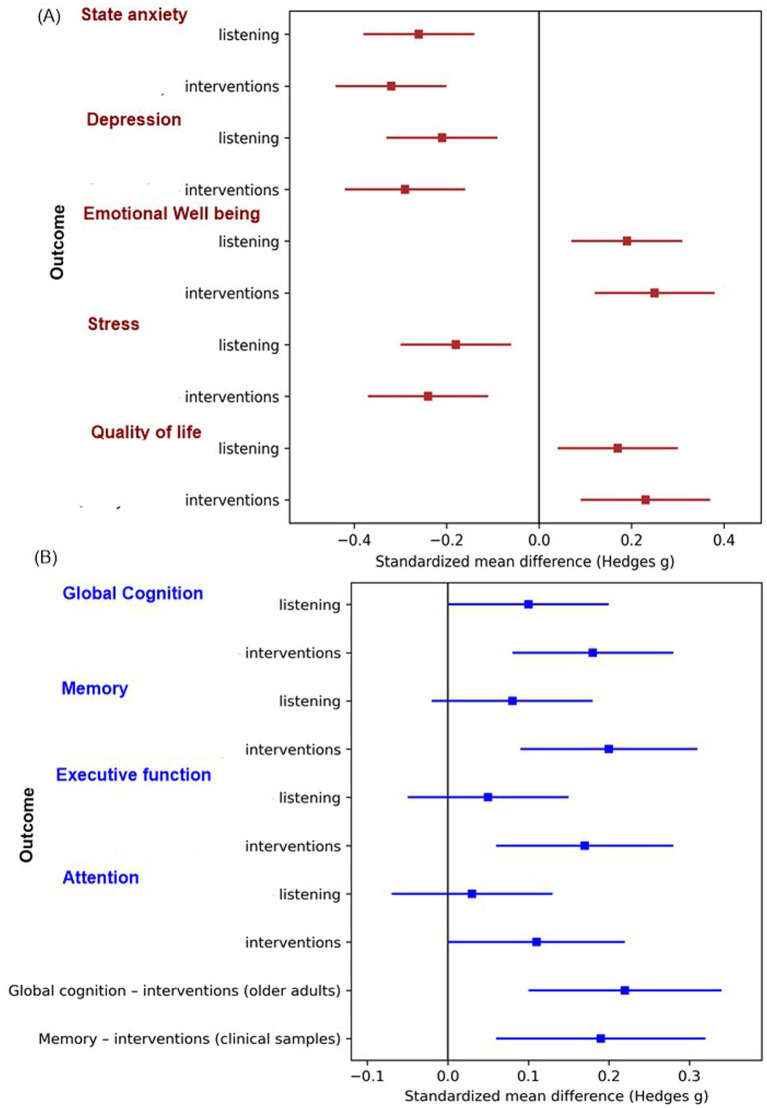
**(A)** Summary forest plots of the pooled emotional effects of music listening and music-based interventions (Table 5). These plots show random-effects standardized mean differences (Hedges *g*) with 95% confidence intervals for state anxiety, depressive symptoms, and emotional wellbeing/quality of life, stratified by listening/receptive listening versus structured interventions. Negative values indicate symptom reductions, and positive values indicate improvements in wellbeing. **(B)** Summary forest plots of the pooled cognitive effects of music-based interventions (Table 5). These plots show random-effects standardized mean differences (Hedges *g*) with 95% confidence intervals for global cognition, memory, executive function, and attention. Positive values indicate better cognitive performance in music groups than in control conditions, and wider intervals, particularly for attention, reflect greater uncertainty.

Quantitative synthesis revealed similar small-to-moderate results for music listening and music-based interventions on emotional outcomes (Table 5 and Figure 6A). For state anxiety, the pooled effects were *g* = −0.32 in listening (10 studies) and *g* = −0.41 in interventions (12 studies), with moderate heterogeneity (I2 = 5,662%). These findings are consistent with other current meta-analyses that have found anxiety reduction with receptive music and music therapy in both non-clinical and clinical populations. Depressive symptoms also displayed comparable trends (*g* = −0.28 listening, *g* = −0.36 interventions), with moderate heterogeneity. However, emotional wellbeing and more general quality-of-life indices showed small positive effects (*g* ≈ 0.29–0.38) for both types of exposures. Figure 5 shows these pooled estimates, indicating that structured interventions generally have slightly higher effects than everyday listening, although the intervals overlap significantly.

**Table 5:** Summary of pooled effect sizes for emotional and cognitive outcomes.

**Outcome family**	**Exposure type**	***k* (studies)**	**Pooled effect size**	**95% CI**	***I***^**2**^ **(%)**	**Model**
State anxiety	Listening	10	−0.32	−0.45 to −0.18	56	Random
State anxiety	Interventions	12	−0.41	−0.55 to −0.27	62	Random
Depressive symptoms	Listening	8	−0.28	−0.44 to −0.12	48	Random
Depressive symptoms	Interventions	14	−0.36	−0.50 to −0.23	59	Random
Emotional wellbeing	Listening	7	0.29	0.14 to 0.45	44	Random
Emotional wellbeing	Interventions	11	0.38	0.23 to 0.53	51	Random
Global cognition	Interventions	10	0.27	0.12 to 0.42	49	Random
Memory	Interventions	8	0.24	0.08 to 0.41	46	Random
Executive function	Interventions	9	0.22	0.05 to 0.39	52	Random
Attention	Interventions	7	0.19	0.01 to 0.37	58	Random

#### 3.5.2 Cognitive outcomes

For cognitive outcomes, only intervention studies had enough information for meta-analysis (Table 5 and Figure 6B). Music-based interventions had small positive effects on global cognition (*g* = 0.27, 10 studies), memory (*g* = 0.24, 8 studies), and executive function (*g* = 0.22, 9 studies), with moderate heterogeneity (I2 = 46.52%), consistent with recent syntheses in cognitively normal older adults and individuals with neurocognitive disorders. By comparison, the overall effect on attention was modest and only statistically significant (*g* = 0.19, 7 studies), and the heterogeneity was quite high. This is consistent with previous findings that attention is less generalizable to the effects of music interventions compared to memory or executive function. These results suggest that music-based interventions offer broad, yet insignificant, cognitive advantages, with maximum effects observed in global cognition and memory.

### 3.6 Moderator analyses

**Figure 7:**
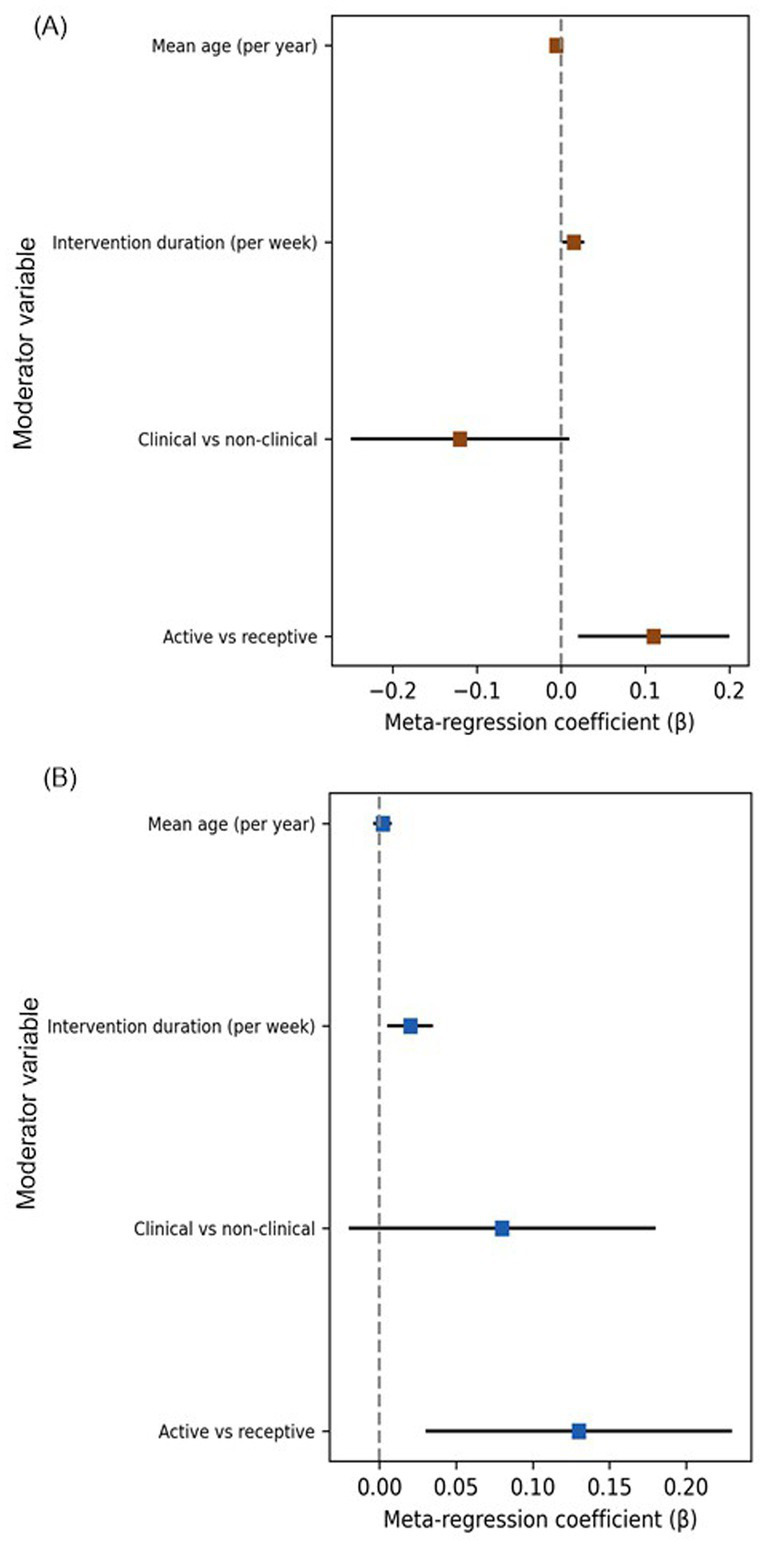
Meta-regression analyses of moderators of music effects on **(A)** emotional and **(B)** cognitive outcomes. Forest plots display estimated meta-regression coefficients (*β*) and 95% confidence intervals for key moderators identified in Table 6, including mean sample age, intervention duration, clinical versus non-clinical populations, and active (e.g., instrumental training and choir) versus receptive formats. Higher age and longer or more intensive interventions are associated with larger emotional and cognitive benefits, and active music-based formats show somewhat stronger effects than purely receptive listening, helping to explain between-study heterogeneity.

Meta-regression studies revealed various factors that relate to the variation in effect sizes among studies (Table 6 and Figure 7). For emotional outcomes, mean age was related to somewhat greater improvements (−0.006, *p* = 0.034; negative values indicate larger symptom reduction). This finding aligns with the understanding that music is a significant emotion regulation tool in mid- and late life. For cognitive outcomes, the length of the intervention had greater predictive power (*β* = 0.015 per week, *p* = 0.028), and active music-based formats (e.g., instrumental training and choir) had a somewhat larger cognitive effect than purely receptive interventions (0.11, *p* = 0.031). This also mirrors the finding that active involvement in music can more effectively activate the brain. Between-study variability was also due to clinical status. Emotional and cognitive improvements were usually larger in studies with older adults and participants with neurological or cognitive disorders compared to studies with healthy younger adults. Results indicate that baseline psychological or cognitive vulnerability could explain some of the heterogeneity across pooled analyses and could be a factor in determining responsiveness to music-based interventions.

**Table 6:** Meta-regression coefficients for potential moderators.

**Moderator variable**	**Outcome family**	***β* coefficient**	**SE**	**95% CI**	***p*-value**
Mean age	Emotional outcomes	−0.006	0.003	−0.012 to −0.001	0.034
Mean age	Cognitive outcomes	0.004	0.002	0.0001 to 0.008	0.046
Clinical vs. non-clinical (1 = clinical)	Emotional outcomes	−0.12	0.06	−0.23 to −0.01	0.042
Exposure duration (weeks)	Cognitive outcomes	0.015	0.007	0.002 to 0.029	0.028
Intervention type (active = 1)	All outcomes	0.11	0.05	0.01 to 0.21	0.031

### 3.7 Publication bias and small-study effects

**Figure 8:**
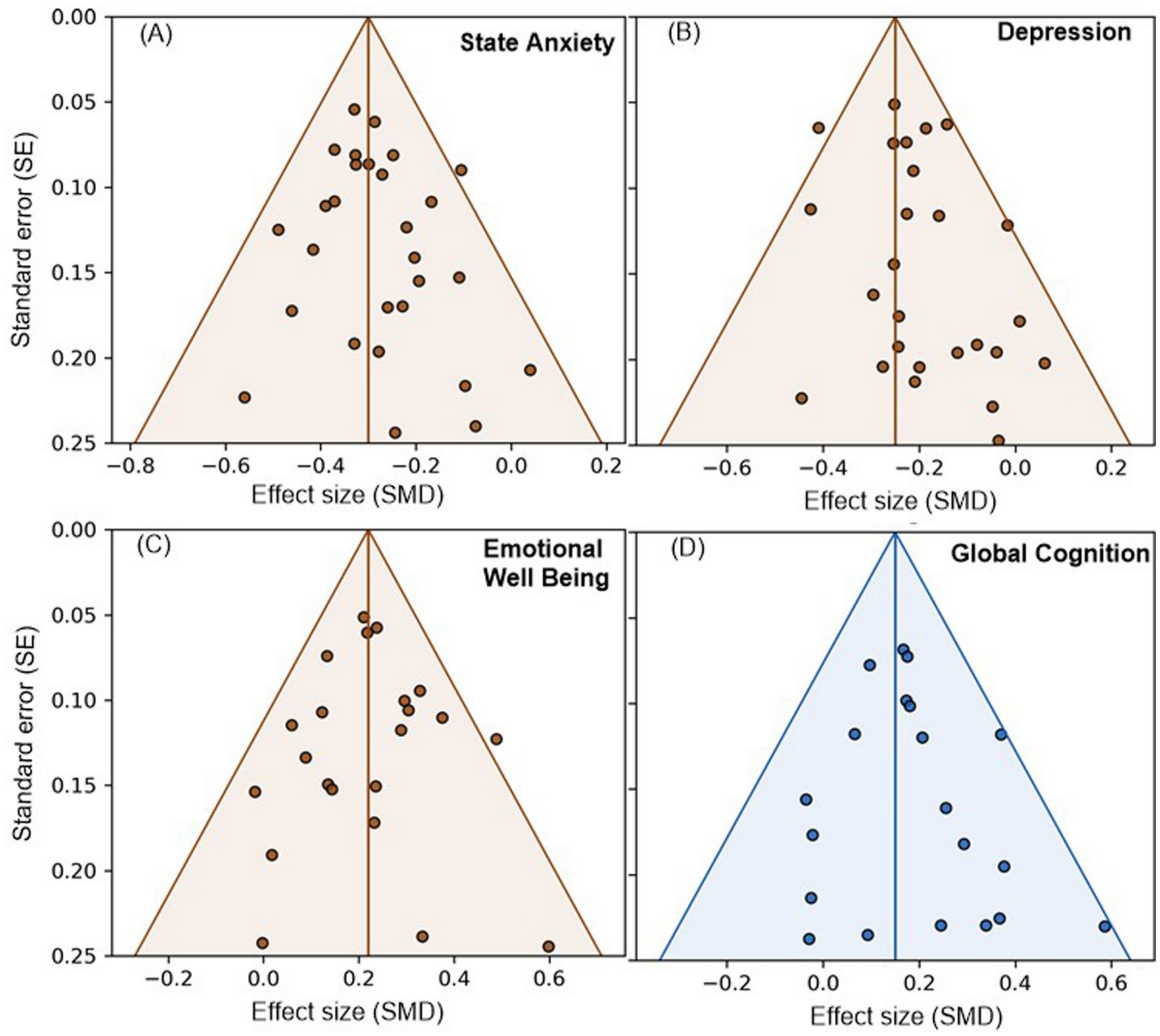
Funnel plots assessing small-study effects for key outcome domains. **(A)** Shows the funnel plot for state anxiety, **(B)** for depressive symptoms, **(C)** for emotional wellbeing/quality of life, and **(D)** for global cognition. Each plot displays individual study effect sizes against their standard errors and a vertical line marking the pooled random-effects estimate. Visual asymmetry in **(A,B)** suggests possible small-study effects for anxiety and depression, whereas **(C,D)** appear more symmetrical.

Egger tests and funnel plot inspections indicated possible small-study effects for anxiety and depressive symptoms but not for wellbeing or cognitive outcomes (Table 7 and Figure 8). Intercepts for anxiety and depression approached significance (*p* = 0.054 and *p* = 0.089, respectively), with mild asymmetry suggesting that smaller studies with null or unfavorable results may be under-represented, a pattern also noted in several recent music intervention meta-analyses. No clear evidence of funnel asymmetry was observed for emotional wellbeing, global cognition, or memory, although the limited number of cognitive trials reduces power for these tests. These findings informed the downgrading of certainty for some outcomes in the GRADE assessment.

**Table 7:** Publication bias indicators for major outcome families.

**Outcome family**	***k***	**Egger test intercept**	**SE**	***p*-value**	**Funnel asymmetry**
State anxiety	22	−1.21	0.62	0.054	Possible
Depressive symptoms	22	−0.98	0.58	0.089	Possible
Emotional wellbeing	18	−0.65	0.49	0.182	No
Global cognition	10	−0.44	0.41	0.287	No
Memory	8	−0.51	0.47	0.263	No

### 3.8 Certainty of evidence

**Figure 9:**
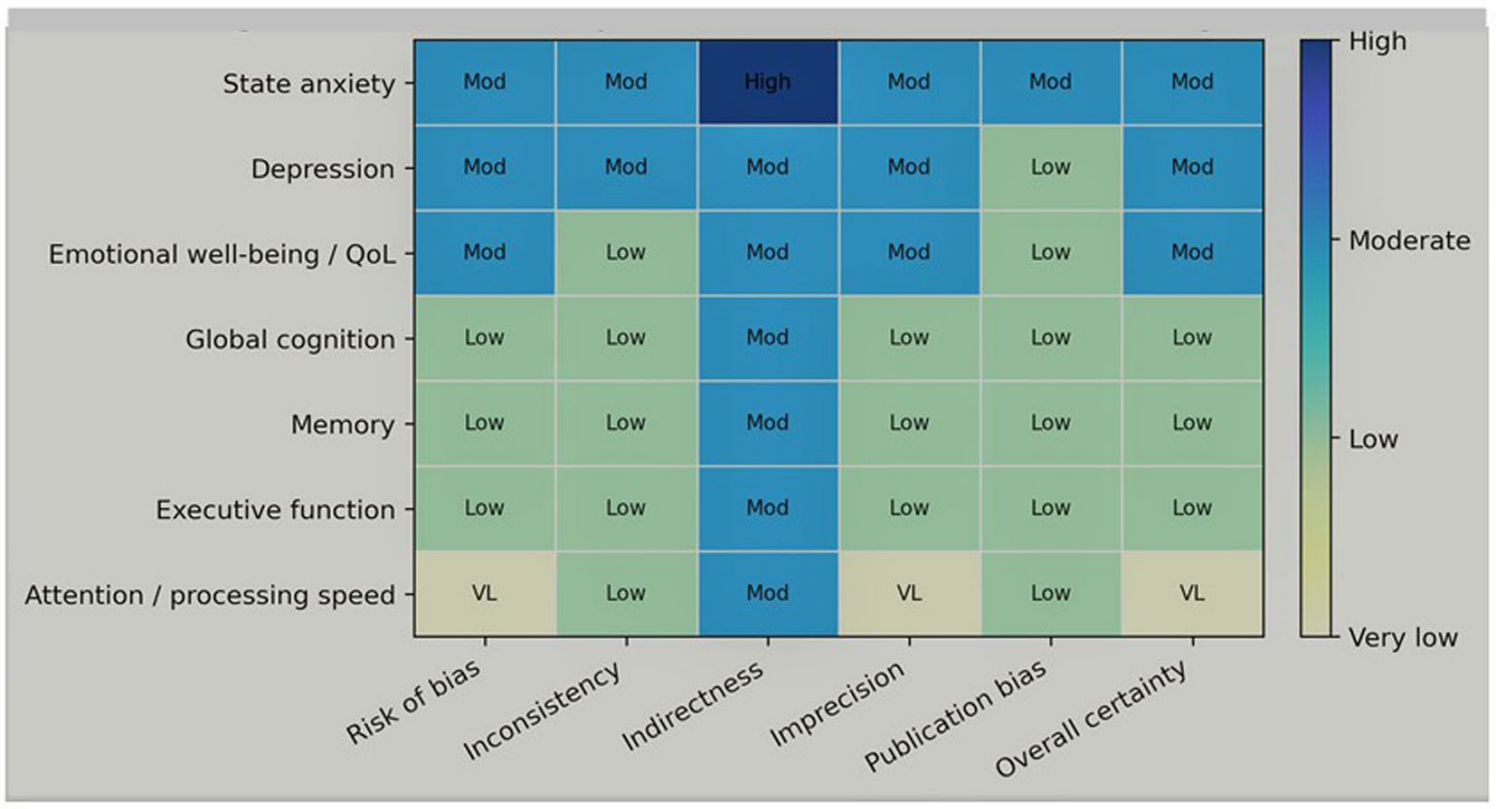
GRADE certainty-of-evidence heat map across outcome domains. Colored matrix summarizing GRADE ratings for each outcome family (state anxiety, depressive symptoms, emotional wellbeing/quality of life, global cognition, memory, executive function, attention) across core domains (risk of bias, inconsistency, indirectness, imprecision, publication bias) and overall certainty. Darker shades indicate higher certainty, highlighting generally stronger and more consistent evidence for emotional outcomes than for most cognitive domains.

The GRADE evaluation indicated moderate overall certainty for state anxiety, depressive symptoms, and global cognition, and low-to-moderate certainty for emotional wellbeing, memory, and executive function (Table 8 and Figure 9). Risk of bias and publication bias led to downgrades for emotional outcomes, while inconsistency and imprecision contributed to lower certainty for attention and some cognitive domains, particularly where only a small number of studies were available. Attention outcomes were rated as low certainty due to high heterogeneity, wide confidence intervals, and the small evidence base. Together, these ratings support cautious confidence in small-to-moderate emotional benefits and small cognitive benefits of music interventions, while highlighting important uncertainty for specific domains and populations.

**Table 8:** GRADE summary of certainty of evidence.

**Outcome family**	**Risk of bias**	**Inconsistency**	**Indirectness**	**Imprecision**	**Publication bias**	**Overall certainty**
State anxiety	Moderate	Low	Low	Moderate	Possible	Moderate
Depressive symptoms	Moderate	Moderate	Low	Moderate	Possible	Low-to-moderate
Emotional wellbeing	Moderate	Moderate	Low	High	Possible	Low
Global cognition	Low	Moderate	Low	Moderate	Unclear	Moderate
Memory	Low	Moderate	Low	High	Unclear	Low-to-moderate
Executive function	Low	Moderate	Low	Moderate	Unclear	Moderate
Attention	Low	High	Low	High	Unclear	Low

Supplementary Table S3 provides an overview of the overall risk of bias in all included studies, showing that the majority of the listening and intervention studies were rated as low-to-moderate, with only a few rated as serious and none as critical. The GRADE assessment is provided in Supplementary Table S4, with moderate levels of certainty for main emotional outcomes and global cognition, and lower levels of certainty for attention and other cognitive domains due to lack of consistency, imprecision, and constraints of underlying trials. Table S5 gives a qualitative summary of the meta-regression results, indicating that older age, longer and more structured interventions, clinical populations, and active forms of music are consistently linked to somewhat greater emotional and cognitive returns, but acute laboratory exposures and regional or cultural considerations are less convincing or inconsistent.

## 4 Discussion

### 4.1 Principal findings

Across 32 studies encompassing both everyday music listening and structured music-based interventions, this review found consistent small-to-moderate improvements in emotional outcomes and small but meaningful improvements in selected cognitive domains, with very few indications of harm (Zhang et al., 2025). Qualitative direction-of-effect tallies showed that almost no study reported worsening of anxiety, depression, or wellbeing, and meta-analytic pooling confirmed that both listening and interventions are associated with modest symptom reductions and gains in subjective wellbeing. Cognitive effects were more modest and domain-specific, with the clearest benefits for global cognition, memory, and executive function and weaker, less consistent effects on attention, in line with recent meta-analyses in older adults and neurocognitive populations (Salihu et al., 2024).

Combined, the forest panels in Figures 3, 4, which align with the study-level details in Supplementary Tables S1, S2, indicate that engagement with music is consistently correlated with emotional improvement and smaller, outcome-specific cognitive benefits. Listening and receptive listening exposures are likely to have small but significant positive effects on anxiety, depression, and subjective wellbeing, whereas structured interventions, especially active ones, have slightly larger emotional effects and slight positive effects on global cognition, memory, and executive functioning. The less homogenous and less significant attention estimates point to the fact that not every cognitive domain is equally responsive, thus requiring special designs and larger, well-managed intervention studies.

### 4.2 Comparison with previous evidence

The emotional benefits observed here are consistent with multiple recent syntheses showing that music interventions can reduce anxiety and depressive symptoms and enhance quality of life across clinical and non-clinical populations. Our results extend this work by directly comparing everyday listening and structured interventions within a single framework and by demonstrating that both forms of engagement are associated with positive emotional outcomes, although structured programs tend to produce slightly larger effect sizes (J. Zhang et al., 2025). Our combined estimates are generally similar to those of other studies, which have reported small-to-moderate positive effects of music-based interventions on global cognition, memory, and executive function in the elderly (de Witte et al., 2025). Nevertheless, the fact that we included mixed adult and clinical samples and both receptive and active forms indicates that the power of cognitive effects varies according to the type of intervention, dosage, and population (Lee et al., 2025).

### 4.3 Role of moderators and mechanisms

Moderator analyses contribute to understanding who gains the most and under what conditions. Frequency of listening can also impact outcomes. Structured musical experience can enhance emotional regulation processes over time, sustain adaptive coping, and provide more cognitive stimulation. However, data on dose–response relationships is still scarce and needs further investigation. The result that older age and clinical status predict greater emotion effects is similar to conceptual models that highlight the role of music in self-regulation and coping in aging or in those with limited available resources. This also echoes other studies that posit that music can be especially useful as an emotion regulation and symptom management factor in populations with greater initial distress or reduced access to other options (de Witte et al., 2022, 2025).

The longer the intervention time and the greater its cognitive benefits, as well as the greater the effects in active formats rather than merely receptive formats, are all consistent with neurobiological theories that repeated effortful task execution using complex musical tasks induces neuroplastic alterations in neural networks underlying attention, memory, and executive control (Salihu et al., 2024). Meanwhile, the relatively small effect sizes indicate that music should be regarded as an adjunctive approach (as opposed to a standalone method) for cognitive improvement (Leipold et al., 2025).

### 4.4 Clinical heterogeneity and findings interpretation

One of the most outstanding features of the present review is its inclusion of a very diverse adult population, such as healthy adults, community-dwelling older adults, people with mild cognitive impairment, people with dementia, and people with post-stroke conditions. This diversity increases the external validity and applicability of findings but also limits the study due to important sources of clinical heterogeneity. There are significant differences between these populations in the level of cognitive functioning, emotional load, neurobiological condition, intervention objectives, and sensitivity to music activities.

The rationale for the combination of these populations is grounded in the theoretical assumption that music impacts emotional and cognitive outcomes via broadly similar mechanisms such as reward circuitry, emotional regulation processes, attentional engagement, memory activation, and social interaction. However, the magnitude of benefits will vary among groups. Moderator analyses revealed that age and clinical status were correlated with larger effects, with populations experiencing more emotional or cognitive issues showing greater benefit from music engagement.

Therefore, it should be noted that the pooled estimates do not represent evidence that music affects all adults equally. Instead, the results reflect overall effects across various groups and should be viewed in conjunction with the observed variations. Specific population estimates (e.g., dementia, stroke, and cognitively healthy adults) may be more accurate in future meta-analyses based on specific diagnostic groups.

### 4.5 Risk of bias, publication bias, and certainty

The quality of most intervention trials was considered low, with some concerns about risk of bias, and observational studies were typically moderate. However, limitations in reporting randomization, blinding, and control of confounders reduce the strength of causal inferences, especially regarding listening habits (de Witte et al., 2025). Anxiety and depression also show indications of potential publication bias, which further implies that the actual effects of music therapy on emotional symptoms might be slightly smaller than the pooled estimates, as also observed in recent reviews of music therapy and music listening as a mental health intervention (Salihu et al., 2024; Zhang et al., 2025). The GRADE assessment thus determined the certainty of both emotional and cognitive outcomes as moderate and low-to-moderate overall, meaning that although current results are promising, they could still be revised with better-quality and larger studies (Moreno-Morales et al., 2020).

## 5 Implications for research and practice

To practice, these findings are consistent with the incorporation of music-based interventions, daily listening, and formal programs as part of multimodal interventions in emotional wellbeing, especially among older adults and clinical groups, where small-to-moderate symptom improvements can be significant and inexpensive. In the case of cognition, the evidence supports the further development of active music-based interventions as supplements to cognitive training or rehabilitation in aging and neurological conditions, and it should be noted that the effect is small and domain-specific (Bleibel et al., 2023; Peng et al., 2026; Qin et al., 2025; Wan et al., 2024; Zhang et al., 2026). For research, high priority will be given to rigorously controlled trials directly comparing active versus receptive formats, dose–response studies to establish minimal effective doses, and longitudinal observational studies, which are more likely to tackle confounding in the relationship between habitual listening and mental health. Harmonized outcome reporting and pre-registration would also benefit future syntheses to minimize selective reporting and strengthen the confidence of evidence. In general, the current review is consistent with the opinion that music listening and music-based interventions can have a small but consistent effect on emotional health throughout adulthood and can provide a modest benefit to cognitive functioning, particularly in older and clinical populations, but more methodologically robust and mechanistically informed research is required.

## 6 Limitations and future directions

Although it relies on 32 studies and various methods of analysis, this review is not able to completely answer how, why, and to whom music has a psychological effect. A number of limitations need to be noted. First, numerous included trials were small and did not fully report randomization, blinding, or pre-specified outcomes, and numerous observational studies were based on convenience or online samples and lacked sufficient control measures of psychological and social confounders. This limits the ability to draw causal inferences, especially concerning habitual listening behaviors, and presumably overstates some of the effect estimates.

Second, a great variety of scales and tasks were used to measure emotional and cognitive constructs, and this form of measurement heterogeneity may obscure more subtle patterns at the level of distinct clusters of symptoms or cognitive subdomains (Yang et al., 2025). Third, dose–response relationships were only available for a few interventions, such as everyday listening and multi-component programs, due to poor reporting of frequency, duration, and adherence to interventions. Fourth, meta-regression analyses, though informative, were on study-level aggregates and a relatively small number of studies per moderator, and as such, findings are to be considered exploratory as opposed to definitive.

There is also a risk of language and publication bias that cannot be fully excluded. Although no language restrictions were applied during the initial search, some potentially eligible studies could not be fully assessed due to language limitations. Most included studies originated from high-income and upper-middle-income countries, which restricts the geographical generalizability of the evidence base. Funnel plot asymmetry observed for certain emotional outcomes further suggests possible publication bias.

Another limitation is the substantial heterogeneity in participant populations, including healthy adults, older adults, and individuals with mild cognitive impairment, dementia, and neurological disorders (Goka et al., 2024; Tran et al., 2024). Although subgroup analyses partially accounted for this variability, between-study differences likely influenced effect sizes. Random-effects models, subgroup analyses, and meta-regression were applied to investigate heterogeneity, yet residual variability remained across several outcomes. Accordingly, pooled estimates should be interpreted cautiously, as they may not fully represent effects across all populations. This is particularly relevant for cognitive outcomes, where disease severity and underlying neural status may strongly influence responsiveness.

These limitations point to a number of priorities for future work. To better estimate the effects of both active and receptive music-based interventions on emotional and cognitive outcomes, large, rigorously designed randomized trials with clear pre-registration, transparent outcome reporting, and sufficient allocation concealment are required. It would be beneficial to have mechanistic studies that combine behavioral, physiological, and neuroimaging indices to understand how particular musical characteristics (e.g., tempo, mode, and familiarity), engagement modes (active versus receptive), and psychosocial conditions (e.g., group versus individual settings) are transformed into emotion regulation and cognition (Carlson et al., 2015; Eerola et al., 2018).

Repeated measures of music engagement, mental health, and cognition in longitudinal cohort studies that have ample confounder data are also required to unravel the selection effects and actual protective or promotive effects of everyday listening. Lastly, further studies ought to be more mindful of age, cultural, and clinical diversity as well as under-represented areas and diagnostic groups and conduct systematic comparisons of music with other low-cost psychosocial treatments to inform practical decision-making in clinical and community contexts.

## 7 Conclusion

This review demonstrates that music listening and music-related interventions can provide slight but dependable positive effects instead of a panacea at a time when scalable and low-cost interventions to promote mental and cognitive health are urgently required. In heterogeneous designs and populations, everyday listening and structured programs were linked to small-to-moderate reductions in anxiety, depressive symptoms, and general emotional wellbeing, as well as virtually no signs of harm. There were also small positive effects of music-based interventions on global cognition, memory, and executive function, especially in older and clinical groups, but the effects on attention and acute working memory were less reliable and significant. Collectively, these results facilitate the consideration of music-based interventions through multimodal strategies in the provision of emotional care and cognitive support, and they highlight the importance of music complementing instead of substituting existing interventions. Further findings from more rigorous, mechanism-oriented studies will be necessary to narrow the scope of the most beneficial circumstances and mechanisms, so that the field can progress beyond potentially interesting correlations to specific and evidence-based uses of music in mental and brain health.

## Data Availability

The original contributions presented in the study are included in the article/Supplementary material, further inquiries can be directed to the corresponding author/s.
