# Supplementary material for: Psychological effects of music listening habits on emotional wellbeing and cognitive performance in adults: a systematic review and meta-analysis
Source: Front Psychol. 2026 Jul 9;17:1846437. doi: 10.3389/fpsyg.2026.1846437 (PMC13391587; doi:10.3389/fpsyg.2026.1846437)
Supplement: Supplementary file 1 [file Data_Sheet_1.docx]

**Psychological Effects of Music Listening Habits on Emotional Well-Being and Cognitive Performance in Adults: A Systematic Review and Meta-analysis**

**Table S1. Primary studies evaluating music listening behaviors and their associations with emotional states and cognitive indicators in adults**

| **Author & Year** | **Country / Setting** | **Design** | **Sample (N, Age)** | **Population** | **Music Listening Exposure** | **Comparator / Reference** | **Emotional Outcomes** | **Cognitive Outcomes** |
| --- | --- | --- | --- | --- | --- | --- | --- | --- |
| (Borella et al., 2014) | Italy; lab | Experimental | Young & older adults (as reported) | Healthy younger & older | Music excerpts during WM task | No‑music / alternative sound | Affective ratings | Working memory span |
| (Filippis & Foysal, 2025) | International; cross‑sectional | Survey | Diverse adults (varied N) | General community | Listening habits & mental health | Within study | Anxiety, depression associations | None |
| (Groarke & Hogan, 2016) | Ireland; community | Mixed methods | N = 43 (24 younger; 19 older) | Everyday listeners | Functions of music listening | N/A | Affect regulation categories | None |
| (Groarke & Hogan, 2018) | Ireland; community | Survey validation | N = 1191 (17–66 yrs) | Adults | Adaptive functions scale | N/A | Well‑being correlations | None |
| (Groarke & Hogan, 2019) | Ireland; lab | RCT with control | N = 80 (18–30, 60–81 yrs) | Healthy adults | Self‑chosen music (10 min) | Active control (radio) | Reduced negative affect | None |
| (Hanser et al., 2022) | International survey | Cross‑sectional | N = 2778 (adults) | General public | Self‑reported crying over music episodes | Within‑subject contexts | Emotional crying triggers | None |
| (Hashim et al., 2023) | UK; lab listening | Cross‑sectional listening task | N = 353 online | Online adult sample | Film music excerpts (happy, tender, fearful) | Internal comparisons (emotion conditions) | Visual imagery linked to affect | None |
| (Henry et al., 2021) | UK; community | Online survey | N = 233 (adults) | Adults (COVID‑19 context) | Music use for mood regulation | N/A | Stress, coping emotions | None |
| (Julia et al., 2025) | Indonesia; cross‑sectional | Survey | N = 405 undergraduates | University students | Daily music listening | Within‑study correlations | Emotional well‑being | Academic stress coping |
| (Linnemann et al., 2015) | Germany; daily life EMA | Ecological momentary assessment | N = 55 adults | Everyday adults | Self‑selected daily listening | Non‑listening moments | Stress, affect | None |
| (Randall & Rickard, 2017) | Australia; experience sampling | Experience sampling (mobile app) | N = 327 (mean ~21) | Young adults | Real‑time personal music listening | Within‑participant | Emotional reasons & affect | None |
| (Thoma et al., 2012) | Switzerland/Germany; web survey | Cross‑sectional | N ≈ 1230 (mean ~25) | University students | Habitual music listening behaviour | Within‑sample associations | Emotion regulation | Psychological functioning |
| (Tervaniemi et al., 2021) | Finland; mixed contexts | Exploratory within‑subjects | Adults (exact N reported) | Everyday & lab listeners | Self‑selected favoured & neutral music at home vs lab | Home vs lab condition | Emotional state ratings; cortisol | None |
| (Vincenzi et al., 2022) | Italy/UK/Canada; lab | Quasi‑experimental | N = 132 older adults | Older adults | Happy/sad music vs spoken word | Spoken‑word control | Arousal & mood | Working memory, flexibility, fluency |
| (Van den Tol & Edwards, 2015) | UK; community | Cross‑sectional survey | Adults (exact N not abstract) | General adult | Listening to sad music | N/A | Mood repair, sadness relief | None |
| (Zhu et al., 2025) | China; large survey | Cross‑sectional | National sample (2010–2023) | General population | Music listening frequency | Non‑listeners | Subjective well‑being | None reported |

**Table S2. Consolidated dataset of validated studies investigating emotional and cognitive responses to music listening and music-based interventions across adult populations**

| **Author & Year** | **Country / Setting** | **Design** | **Sample (N, Age)** | **Population** | **Music‑Based Intervention** | **Comparator / Control** | **Emotional Outcomes** | **Cognitive / Neuropsychiatric Outcomes** |
| --- | --- | --- | --- | --- | --- | --- | --- | --- |
| (Bugos, 2019) | USA; community | RCT | N = 33; age 60–85 | Healthy older adults | Bimanual coordination intervention | No intervention | Mood | Executive functions |
| (Chan et al., 2010) | Taiwan; community / institution | RCT | N = 47; ≥60 yrs | Older adults | Relaxing music listening sessions | Standard care | Depression, sleep quality | None / general cognition |
| (Gök Ugur et al., 2017) | Hong Kong; nursing home | RCT | N = 60; ≥65 yrs | Nursing‑home resident | Group music intervention | Standard care | Depression, physiological measures | None / general |
| (Cooke et al., 2010) | Australia; dementia | RCT | N = 47; ≥80 yrs | Older adults with dementia | Live music with reading | Reading activity | Depression, belonging, self‑esteem | Behavioral engagement |
| (Dorris et al., 2021) | International | Systematic review & meta‑analysis | Multi‑study | MCI/dementia | Choir, group music, receptive music | Standard care | Depression, QoL, BPSD | Global cognition |
| (Cheung et al., 2018) | Hong Kong; dementia | RCT | N = 63; mean ≈ 85 yrs | Moderate dementia | Music‑with‑movement | Standard/social activities | Mood, anxiety | MMSE + domain cognition |
| (Cheung et al., 2020) | Hong Kong; dementia | RCT (3‑arm) | N = 165; mean ≈ 85 yrs | Moderate dementia | Movement vs listening vs social activity | Multi‑arm | Agitation, BPSD, mood | Cognition (if assessed) |
| (Johnson et al., 2020) | USA; community centers | Cluster RCT | N = 390; mean ~71 yrs | Community older adults | Weekly choir | Usual programs | Well‑being, loneliness | Limited cognition |
| (Kim & Kang, 2021) | South Korea; nursing homes | RCT | N = 40; ≥65 yrs | Elderly resident | Music + rhythmic exercise | Usual activity | Depression, anxiety, life satisfaction | MMSE‑K |
| (MacRitchie et al., 2020) | Europe; community | RCT / feasibility | N = 46; ≥60 yrs | Healthy older adults | Music instrument or group music sessions | Waitlist | Well‑being, social connection | Exploratory cognitive indicators |
| (Guétin et al., 2009) | Italy; dementia | RCT | N = 50; mean ≈ 82 yrs | Alzheimer’s dementia | Structured music therapy | Standard care | Anxiety, depression | Cognitive status |
| (Sung et al., 2012) | Italy; nursing homes | RCT | N = 60; mean ≈ 84 yrs | Dementia | Percussion‑based music intervention | Standard care | Anxiety, agitation, mood (BPSD) | MMSE |
| (Sarkamo et al., 2008) | Finland; post‑stroke rehab | RCT | N = 60; recent stroke | Stroke survivors | Daily music listening program | Audiobook / usual care | Mood, depressive symptoms | Memory, attention |
| (Seinfeld et al., 2013) | Spain; community | RCT | N = 64; mean age ≈ 67 | Healthy older adults | Piano‑training program | Waiting list | Depressive symptoms, mood, QoL | Memory, executive function |
| (Xue et al., 2023) | China; MCI + depression | RCT | N = 83; ≥60 yrs | Older adults with MCI + depression | Receptive music therapy | Standard care | Depression, mood, QoL | Cognitive function |
| (Yu et al., 2022) | Taiwan; nursing homes | RCT | N = 63; ≥65 yrs | Elderly resident | Group music sessions | Usual care | Depression | None |

# ****Supplementary Table S3. Risk of Bias Overview (ROBINS-I + RoB 2.0 Combined Summary)****

| **Study Type** | **Low Risk** | **Moderate** | **Serious** | **Critical** |
| --- | --- | --- | --- | --- |
| Listening (n=16) | 6 | 8 | 2 | 0 |
| Interventions (n=16) | 10 | 5 | 1 | 0 |

# ****Supplementary Table S4. GRADE Summary of Certainty of Evidence****

| **Outcome Category** | **Number of Studies** | **Consistency** | **Precision** | **Risk of Bias** | **Certainty Level** |
| --- | --- | --- | --- | --- | --- |
| Emotional outcomes (all) | 32 | High | Moderate | Moderate | Moderate |
| Anxiety | 20 | High | Moderate | Moderate | Moderate |
| Depression | 22 | High | Moderate | Moderate | Moderate |
| Global cognition | 11 | Moderate | Low | Low | Moderate |
| Memory | 12 | Moderate | Low | Low | Moderate |
| Executive function | 10 | Moderate | Low | Low | Low-to-moderate |
| Attention | 6 | Low | Low | Low | Low |

# ****Table S5. Qualitative Meta-Regression Summary for Studies Reporting Standardized Coefficients****

| **Predictor** | **Number of Studies Reporting Coefficients** | **Direction of Association** | **Strength of Evidence** |
| --- | --- | --- | --- |
| Older age | 6 | Stronger emotional improvements | Moderate |
| Longer intervention duration | 10 | Stronger emotional and cognitive improvements | Moderate |
| Structured weekly intervention | 8 | Stronger emotional benefits | Moderate |
| Acute laboratory exposure | 7 | Small transient effects | Low |
| Clinical vs healthy population | 9 | Larger improvements in clinical groups | Moderate |
| Mode of music (active vs passive) | 12 | Active > passive for cognition | Moderate |
| Cultural/region differences | 18 | No consistent pattern | Low |

**References**

Borella, E., Carretti, B., Grassi, M., Nucci, M., & Sciore, R. (2014). Are age-related differences between young and older adults in an affective working memory test sensitive to the music effects? *Frontiers in Aging Neuroscience*, *6*. https://doi.org/10.3389/fnagi.2014.00298

Bugos, J. A. (2019). The Effects of Bimanual Coordination in Music Interventions on Executive Functions in Aging Adults. *Frontiers in Integrative Neuroscience*, *13*. https://doi.org/10.3389/fnint.2019.00068

Chan, M. F., Chan, E. A., & Mok, E. (2010). Effects of music on depression and sleep quality in elderly people: A randomised controlled trial. *Complementary Therapies in Medicine*, *18*(3–4), 150–159. https://doi.org/10.1016/j.ctim.2010.02.004

Cheung, D. S. K., Lai, C. K. Y., Wong, F. K. Y., & Leung, M. C. P. (2018). The effects of the music-with-movement intervention on the cognitive functions of people with moderate dementia: a randomized controlled trial. *Aging & Mental Health*, *22*(3), 306–315. https://doi.org/10.1080/13607863.2016.1251571

Cheung, D. S. K., Lai, C. K. Y., Wong, F. K. Y., & Leung, M. C. P. (2020). Is music-with-movement intervention better than music listening and social activities in alleviating agitation of people with moderate dementia? A randomized controlled trial. *Dementia*, *19*(5), 1413–1425. https://doi.org/10.1177/1471301218800195

Cooke, M., Moyle, W., Shum, D., Harrison, S., & Murfield, J. (2010). A Randomized Controlled Trial Exploring the Effect of Music on Quality of Life and Depression in Older People with Dementia. *Journal of Health Psychology*, *15*(5), 765–776. https://doi.org/10.1177/1359105310368188

Dorris, J. L., Neely, S., Terhorst, L., VonVille, H. M., & Rodakowski, J. (2021). Effects of music participation for <scp>mild cognitive impairment</scp> and dementia: A systematic review and <scp>meta‐analysis</scp>. *Journal of the American Geriatrics Society*, *69*(9), 2659–2667. https://doi.org/10.1111/jgs.17208

Filippis, R. de, & Foysal, A. Al. (2025). Associations between Music Listening Habits and Mental Health: A Cross-Sectional Analysis. *OALib*, *12*(04), 1–29. https://doi.org/10.4236/oalib.1113196

Gök Ugur, H., Yaman Aktaş, Y., Orak, O. S., Saglambilen, O., & Aydin Avci, İ. (2017). The effect of music therapy on depression and physiological parameters in elderly people living in a Turkish nursing home: a randomized-controlled trial. *Aging & Mental Health*, *21*(12), 1280–1286. https://doi.org/10.1080/13607863.2016.1222348

Groarke, J. M., & Hogan, M. J. (2016). Enhancing wellbeing: An emerging model of the adaptive functions of music listening. *Psychology of Music*, *44*(4), 769–791. https://doi.org/10.1177/0305735615591844

Groarke, J. M., & Hogan, M. J. (2018). Development and Psychometric Evaluation of the Adaptive Functions of Music Listening Scale. *Frontiers in Psychology*, *9*. https://doi.org/10.3389/fpsyg.2018.00516

Groarke, J. M., & Hogan, M. J. (2019). Listening to self-chosen music regulates induced negative affect for both younger and older adults. *PLOS ONE*, *14*(6), e0218017. https://doi.org/10.1371/journal.pone.0218017

Guétin, S., Portet, F., Picot, M. C., Pommié, C., Messaoudi, M., Djabelkir, L., Olsen, A. L., Cano, M. M., Lecourt, E., & Touchon, J. (2009). Effect of Music Therapy on Anxiety and Depression in Patients with Alzheimer’s Type Dementia: Randomised, Controlled Study. *Dementia and Geriatric Cognitive Disorders*, *28*(1), 36–46. https://doi.org/10.1159/000229024

Hanser, W. E., Mark, R. E., & Vingerhoets, A. J. J. M. (2022). Everyday crying over music: A survey. *Musicae Scientiae*, *26*(3), 516–537. https://doi.org/10.1177/1029864920981110

Hashim, S., Stewart, L., Küssner, M. B., & Omigie, D. (2023). Music listening evokes story-like visual imagery with both idiosyncratic and shared content. *PLOS ONE*, *18*(10), e0293412. https://doi.org/10.1371/journal.pone.0293412

Henry, N., Kayser, D., & Egermann, H. (2021). Music in Mood Regulation and Coping Orientations in Response to COVID-19 Lockdown Measures Within the United Kingdom. *Frontiers in Psychology*, *12*. https://doi.org/10.3389/fpsyg.2021.647879

Johnson, J. K., Stewart, A. L., Acree, M., Nápoles, A. M., Flatt, J. D., Max, W. B., & Gregorich, S. E. (2020). A Community Choir Intervention to Promote Well-Being Among Diverse Older Adults: Results From the Community of Voices Trial. *The Journals of Gerontology: Series B*, *75*(3), 549–559. https://doi.org/10.1093/geronb/gby132

Julia, J., Supriyadi, T., Sobirjonovna, U. M., Qahramonovna, R. M., Smatillayevna, J. M., Rustam Qizi, Y. L., & Komiljon Kizi, S. A. (2025). Music consumption patterns and their relationship with emotional well-being among university students. *Personality and Individual Differences*, *246*, 113375. https://doi.org/10.1016/j.paid.2025.113375

Kim, H.-S., & Kang, J.-S. (2021). Effect of a group music intervention on cognitive function and mental health outcomes among nursing home residents: A randomized controlled pilot study. *Geriatric Nursing*, *42*(3), 650–656. https://doi.org/10.1016/j.gerinurse.2021.03.012

Linnemann, A., Ditzen, B., Strahler, J., Doerr, J. M., & Nater, U. M. (2015). Music listening as a means of stress reduction in daily life. *Psychoneuroendocrinology*, *60*, 82–90. https://doi.org/10.1016/j.psyneuen.2015.06.008

MacRitchie, J., Breaden, M., Milne, A. J., & McIntyre, S. (2020). Cognitive, Motor and Social Factors of Music Instrument Training Programs for Older Adults’ Improved Wellbeing. *Frontiers in Psychology*, *10*. https://doi.org/10.3389/fpsyg.2019.02868

Randall, W. M., & Rickard, N. S. (2017). Reasons for personal music listening: A mobile experience sampling study of emotional outcomes. *Psychology of Music*, *45*(4), 479–495. https://doi.org/10.1177/0305735616666939

Sarkamo, T., Tervaniemi, M., Laitinen, S., Forsblom, A., Soinila, S., Mikkonen, M., Autti, T., Silvennoinen, H. M., Erkkila, J., Laine, M., Peretz, I., & Hietanen, M. (2008). Music listening enhances cognitive recovery and mood after middle cerebral artery stroke. *Brain*, *131*(3), 866–876. https://doi.org/10.1093/brain/awn013

Seinfeld, S., Figueroa, H., Ortiz-Gil, J., & Sanchez-Vives, M. V. (2013). Effects of music learning and piano practice on cognitive function, mood and quality of life in older adults. *Frontiers in Psychology*, *4*. https://doi.org/10.3389/fpsyg.2013.00810

Sung, H., Lee, W., Li, T., & Watson, R. (2012). A group music intervention using percussion instruments with familiar music to reduce anxiety and agitation of institutionalized older adults with dementia. *International Journal of Geriatric Psychiatry*, *27*(6), 621–627. https://doi.org/10.1002/gps.2761

Tervaniemi, M., Makkonen, T., & Nie, P. (2021). Psychological and Physiological Signatures of Music Listening in Different Listening Environments—An Exploratory Study. *Brain Sciences*, *11*(5), 593. https://doi.org/10.3390/brainsci11050593

Thoma, M. V., Scholz, U., Ehlert, U., & Nater, U. M. (2012). Listening to music and physiological and psychological functioning: The mediating role of emotion regulation and stress reactivity. *Psychology & Health*, *27*(2), 227–241. https://doi.org/10.1080/08870446.2011.575225

Van den Tol, A. J. M., & Edwards, J. (2015). Listening to sad music in adverse situations: How music selection strategies relate to self-regulatory goals, listening effects, and mood enhancement. *Psychology of Music*, *43*(4), 473–494. https://doi.org/10.1177/0305735613517410

Vincenzi, M., Borella, E., Sella, E., Lima, C. F., De Beni, R., & Schellenberg, E. G. (2022). Music Listening, Emotion, and Cognition in Older Adults. *Brain Sciences*, *12*(11), 1567. https://doi.org/10.3390/brainsci12111567

Xue, B., Meng, X., Liu, Q., & Luo, X. (2023). The effect of receptive music therapy on older adults with mild cognitive impairment and depression: a randomized controlled trial. *Scientific Reports*, *13*(1), 22159. https://doi.org/10.1038/s41598-023-49162-6

Yu, A.-L., Lo, S.-F., Chen, P.-Y., & Lu, S.-F. (2022). Effects of Group Music Intervention on Depression for Elderly People in Nursing Homes. *International Journal of Environmental Research and Public Health*, *19*(15), 9291. https://doi.org/10.3390/ijerph19159291

Zhu, W., Ji, Y., & Liu, S. (2025). The relationship between music listening and subjective well-being: evidence from the Chinese General Social Survey (2010–2023). *Frontiers in Psychology*, *16*. https://doi.org/10.3389/fpsyg.2025.1716427
